# Complication inhabituelle de la radiothérapie: la perforation cornéenne: à propos d’un cas

**DOI:** 10.11604/pamj.2016.25.64.9980

**Published:** 2016-10-03

**Authors:** Taha Elghazi, Youssef Omor, Zouheir Hafidi, Amine Eljai, Zakaria Elmoize, Mohammed Afif, Abdellah Amazouzi, Lalla Ouafae Cherkaoui, Rajae Daoudi

**Affiliations:** 1 Université Mohammed V Souissi, Service d’Ophtalmologie A de l’Hôpital des Spécialités, Centre Hospitalier Universitaire, Rabat, Maroc; 2 Université Mohammed V Souissi, Service de Radiologie, Institut National d’oncologie, Rabat, Maroc; 3 Université Mohammed V Souissi, Service de Radiothérapie, Institut National d’oncologie, Rabat, Maroc

**Keywords:** Perforation cornéenne, radiothérapie, kératoplastie transfixiante, Corneal perforation, radiotherapy, penetrating keratoplasty

## Abstract

Les complications oculaires post radiques sont fréquentes au cours de l'irradiation des tumeurs de la tête et du cou. Certaines sont bénignes et transitoires, d'autres peuvent être très graves pouvant mettre en jeu la fonction visuelle. Nous discuterons à travers ce cas rare et inhabituel, les différentes manifestations et complications oculaires et surtout cornéenne de la radiothérapie ainsi que les modalités diagnostiques et thérapeutiques d'une perforation cornéenne qui représente une complication redoutable de la radiothérapie.

## Abstract (en)

Radiation-induced ocular complications are common during radiation therapy for cancers of the head and neck. Some are mild and transient, others can be very serious jeopardizing visual function. This rare and unusual case study aims to highlight the different clinical manifestations and the ocular but especially corneal complications associated with radiation therapy as well as the diagnostic and therapeutic procedures of a corneal perforation which is a serious complication of radiation therapy.

## Introduction

Les complications oculaires de la radiothérapie sont rares mais redoutables, pouvant aller d'une simple irritation conjonctivale, à la nécrose oculaire, en passant par la cataracte, les atteintes rétiniennes, et la neuropathie optique [1]. L'incidence des nécroses cornéennes post-radiques est très faible, et seulement quelques cas ont été rapportés dans la littérature. Nous rapportons dans notre travail, un rare cas de perforation cornéenne isolée, chez un patient traité par radiothérapie pour un carcinome du sinus maxillaire gauche.

## Patient et observation

**Figure 1:**
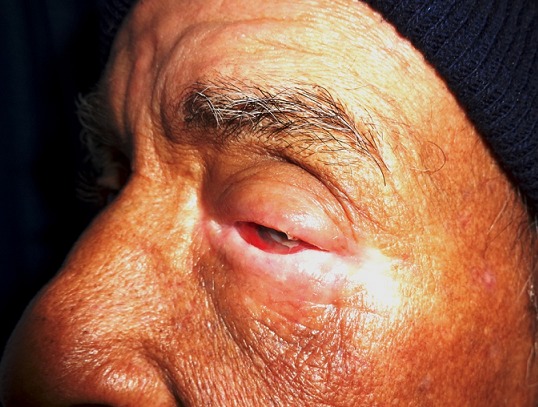
Vue de profil montrant l’amincissement et l’atrophie des paupières

**Figure 2:**
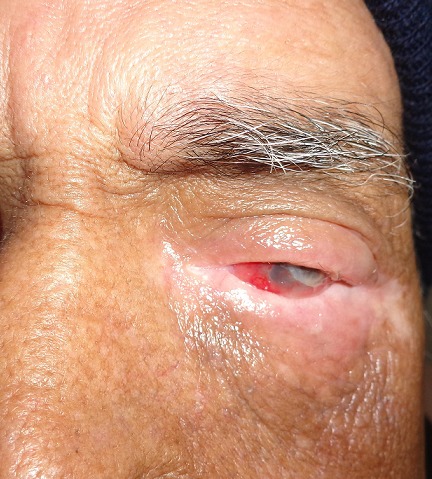
Vue de face montrant la perte des cils palpébraux (madarose)

**Figure 3:**
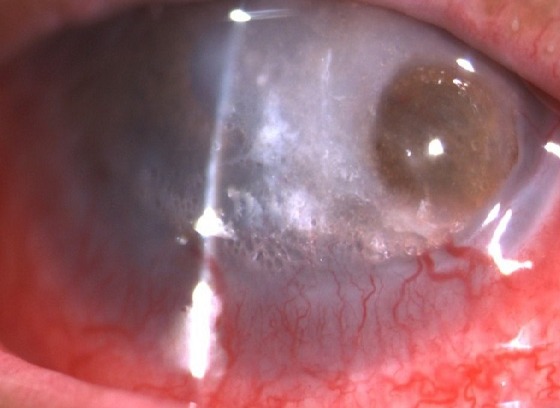
Perforation cornéenne large avec infiltrat stromal diffus prenant toute la cornée

**Figure 4:**
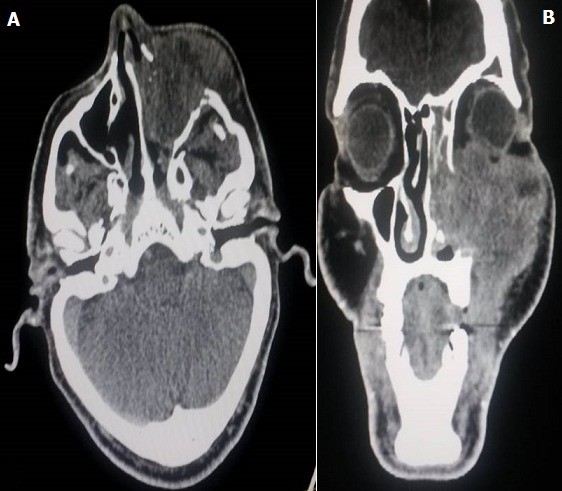
Image scannographique en coupe axiale (a) et coronale(b) montrant la tumeur du sinus maxillaire gauche avec son extension sur l’orbite gauche

Il s'agit d'un patient âgé de 61 ans, qui a été suivi au centre national d'oncologie à Rabat pour tumeur du sinus maxillaire gauche localement avancé. Il a bénéficié 8 mois avant son admission aux urgences ophtalmologiques de séance de radiothérapie à la dose de 70 Gy délivré en 35 fractions, avec fractionnement classique de 2 Gy/fraction, et 6 séances de chimiothérapie à base de cisplatine 40mg/m^2^ hebdomadaire. Il n'y avait pas de notion de traumatisme oculaire. Par ailleurs, le patient a présenté des épisodes de rougeur oculaire à répétition concomitant avec les séances de radiothérapie. L'évolution a été marquée par l'aggravation de la symptomatologie avec présence de douleur et rougeur oculaire associé à une baisse d'acuité visuelle de l'œil gauche. L'examen ophtalmologique trouve une acuité visuelle à perception lumineuse positive au niveau de l'œil gauche. L'examen des annexes montre un amincissement des paupières avec perte des cils palpébraux (madarose) et des secrétions purulentes et mousseuses (Figure 1 et Figure 2). Le segment antérieur note un aspect de perforation cornéenne en temporale de la cornée de 4mm de diamètre horizontal sur 3mm de diamètre vertical colmaté par l'iris entouré d'un infiltrat stromal diffus avec œdème péri-lésionnel et un appel vasculaire en inférieur (Figure 3). La chambre antérieure est très réduite de profondeur. Le reste de l'examen est inaccessible. L'examen de l'œil droit est normal. Un prélèvement systématique a été réalisé. Notre patient a reçu une antibiothérapie empirique à large spectre à base d'une association Vancomycine + ceftazidime en collyre fortifié. Des collyres mouillants ont été prescrits. Le traitement a ensuite été réajusté rapidement vu l'étendu de la perforation. L'évolution a été marquée par une reconstitution d'une petite partie de stroma en gardant une taie cornéenne très étendu. Le patient est en attente d'une greffe de cornée transfixiante. La tomodensitométrie montre bien l'étendu de la tumeur au niveau du sinus maxillaire gauche et son rapport avec l'orbite (Figure 4).

## Discussion

L'œ'il est un organe complexe, composé de plusieurs tuniques de sensibilité différente. Le profil de toxicité oculaire à la radiothérapie est très large [2]. La cataracte représente la complication la plus fréquente et la plus bénigne. La grande sensibilité du cristallin le rend extrêmement sensible, même à des doses de l'ordre de 10-12 Gy. La neuropathie optique et l'atrophie optique constituent des complications redoutables pouvant mettre en jeu le pronostic visuel, et il est recommandé de ne pas dépasser des doses de l'ordre de 50 Gy au niveau de la rétine, et 54 Gy au niveau des nerfs optiques afin de réduire ces risques [3]. Quand à la cornée, il s'agit d'une lentille résistante, et les limites de doses ne sont pas bien précisées dans la littérature. Des doses de l'ordre de 60 Gy délivré en fractionnement classique ont été associées avec des cas de nécrose cornéenne [4]. Chez notre patient, la tumeur était très localement avancée avec extension orbitaire, et ainsi proche de l'œil gauche, le globe oculaire a reçu une dose moyenne de 54 Gy, et certains endroits de la cornée ont reçu des doses supérieures à 60 Gy.

La perte des cils ou madarose constitue l´un des premiers et des plus courants effets indésirables de la radiothérapie. Une desquamation de la peau peut survenir à des doses faibles (10 Gy) et la dermatite qui est plus grave se produit avec des doses plus élevées (40 Gy). On peut assister à des séquelles tardives touchant les paupières comme le trichiasis, les télangiectasies, une hyperpigmentation, une hyperkératose, un entropion, un ectropion, et l´occlusion des points lacrymaux [5].

Les effets aigus de la radiothérapie au niveau cornéen sont rares et se limitent à une simple kératite ponctuée superficielle (KPS) transitoire, qui apparaît vers les 3^ème^ - 4^ème^ semaines de la radiothérapie, et qui se résolve en général quelques semaines après la fin du traitement. La toxicité tardive est rare, et se manifeste souvent par l'apparition d'un ulcère de cornée pouvant aller jusqu'à la nécrose cornéenne. La sévérité de la kératite post radique dépend principalement de la dose reçue au niveau de la cornée et le reste des constituants du globe oculaire. Quant à La sécheresse oculaire causée par l'irradiation de la glande lacrymale, elle peut aggraver les lésions cornéennes. Le tableau clinique initial correspond à une simple rougeur oculaire plus ou moins douloureuse. Une hypoesthésie voire une anesthésie cornéenne s'installe tardivement pouvant se compliquer d'ulcération indolore. L'évolution vers la perforation représente le stade le plus tardif et le plus grave [6].

Le traitement initial des complications cornéennes liées à la radiothérapie vise à éliminer tous les éléments nocifs qui peuvent causer d´autres dommages aux tissus. Des traitements spécifiques dépendent de la gravité de l'atteinte. Des collyres mouillants sans conservateur peuvent être utiles vu le dysfonctionnement lacrymal liés à ces cas. En outre, un traitement par une pommade cicatrisante et / ou par lentille thérapeutique peut être approprié pour certains cas bénins. Concernant la perforation cornéenne qui représente une complication grave, source de morbidité oculaire et de cécité [7, 8], Un traitement chirurgical en urgence est nécessaire pour rétablir l'étanchéité du globe oculaire et prévenir l'endophtalmie. Plusieurs techniques peuvent être utilisées. Certaines sont temporaires, d'autres sont définitives comme la kératoplastie transfixiante [9]. Le choix de la technique appropriée est guidé par la taille, le siège, l'étiologie de la perforation ainsi que les moyens disponibles aux urgences. Les larges perforations comme le cas de notre patient ainsi que les récidives peuvent nécessiter une greffe de cornée transfixiante de première intention. Parfois plusieurs interventions sont nécessaires pour obtenir un succès anatomique et améliorer le pronostic fonctionnel [10].

## Conclusion

La radiothérapie occupe une place majeure dans le traitement des tumeurs de la tête et du cou. Cependant, des techniques de radiothérapie peuvent entraîner dans de rares cas des complications graves sur le plan oculaire. En effet, on peut assister à une perforation cornéenne pouvant mettre en jeu le pronostic fonctionnel de lœil d'où l'intérêt de bien connaitre les mesures de prévention et de traitement des atteintes oculaires à un stade très précoce.
